# Complementary Feeding Practices and Household Food Insecurity Status of Children Aged 6–23 Months in Shashemene City West Arsi Zone, Oromia, Ethiopia

**DOI:** 10.1155/2022/9387031

**Published:** 2022-04-12

**Authors:** Junayde Abdurahmen Ahmed, Kebede Kumsa Sadeta, Kelil Hussen Lembo

**Affiliations:** ^1^Department of of Public Health, School of Health Sciences, Shashemene Campus of Madda Walabu University, Robe, Ethiopia; ^2^Departement of Nursing, School of Health Sciences, Shashemene Campus of Madda Walabu University, Robe, Ethiopia

## Abstract

**Introduction:**

Appropriate infant feeding practices are critical to a child's growth, health, and development during the first 1000 days of life. One in every six children worldwide receives a minimum acceptable diet. According to the EDHS 2016, the status of the minimum acceptable diet was 7 percent among children aged 6–23 months in Ethiopia. The study sought to ascertain the relationship between complementary feeding (CF) indicators and household food insecurity in children aged 6–23 months.

**Methods:**

A systematic sampling method was used to conduct a cross-sectional study of 536 mother-child pairs aged 6–23 months. The 24-hour dietary recall was used to collect data on CF practices using face-to-face interviews with socioeconomic and food security questionnaires. The relationship between complementary feeding indicators and household food insecurity was investigated using logistic regression analysis. The relationship between independent variables and complementary feeding indicators was determined using multivariate logistic regression.

**Results:**

Overall, a total of 67.9% of children received timely introduction of CF and Minimum Meal Frequency (MMF), Minimum Dietary Diversity (MDD), and Minimum Acceptable Diet were met by 61.7%, 42.5%, and 41.7%, respectively. Result of multivariate logistic regression showed there is significant association between household food security with MMF, MDD, and MAD [AOR: 2.02, 95% CI: (1.25–3.24); AOR: 1.55, 95% CI: (1.02–2.36); and AOR: 1.62, 9595% CI: (1.06–2.47)], respectively, while there was no association with introduction of CF [AOR = 0.87, 95% CI: (0.55–1.39)].

**Conclusion:**

This study revealed that the rates of MMF, MDD, and MAD remained low in this study setting. Household socioeconomic status (wealth index, food security status, household income) and child age were found to be among the factors statistically significantly associated with complementary feeding practices indicators.

## 1. Introduction

The first 1000 days of a child's life are crucial for promoting health and development as well as preventing stunting. Inadequate quantities and quality of complementary foods, as well as poor feeding practices and increased infection rates during this period, may be risk factors for stunting in children [1]. Appropriate infant feeding practices are critical for a child's growth, health, and development during the first two years of life [2]. CF refers to a gradual dietary transition involving the introduction of solid and semisolid foods to the infant's diet when breast milk alone is no longer sufficient to meet an infant's nutritional needs [3].

WHO recommends that all children be exclusively breastfed for the first six months of their lives and then receive complementary foods that are nutritionally safe and adequate until they are two years old or older [4]. Malnutrition has been linked to poor breastfeeding and complementary feeding practices of mothers, as well as a high rate of infectious diseases, in the first two years of life [5]. Complementary feeding practices that are insufficient in terms of quality, quantity, and frequency of meals have a negative impact on children's health and growth in the first two years of life [6, 7]. Globally, one in six children receives a minimum acceptable diet [8]. The study found that less than one-fourth of the children aged 6–23 months in developing countries had good consumption quality [8]. WHO shows the effect of feeding practices on the nutritional status of children; about 32% of children under 5 were stunted and 10% were wasted due to poor BF and CF [9].

**Figure 1:**
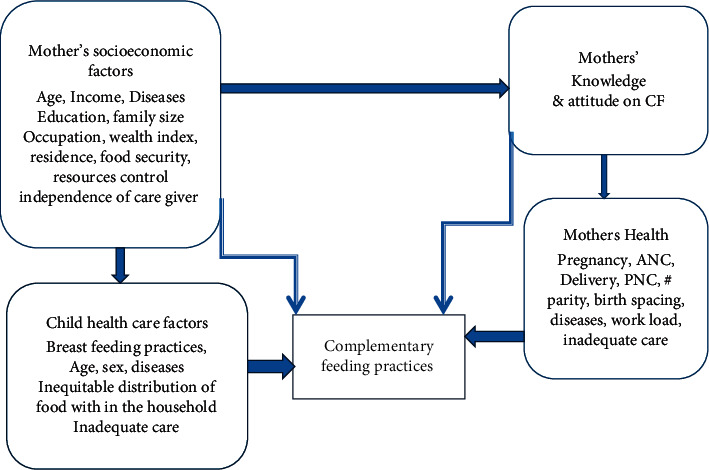
Conceptual framework of complementary feeding practice from the different literature review, 2020. CFP: complementary feeding practices, PNC: postnatal care, and ANC: antenatal care.

Household food insecurity is another important cause of malnutrition that has an effect on vulnerable groups in societies, especially the poor women of reproductive age and children under the age of five as they are at high risk [10]. The 2018 Global Nutrition Report revealed the fact that diets of infant and young children are suboptimal everywhere in all wealth groups from 75.6% in the lowest to 56.7% highest quintile; besides this, 74.6% of children of 6–23 months of age do not have sufficient diet diversity for a healthy diet worldwide [11]. Like most developing countries, improper feeding practices remain a challenging problem in Ethiopia. In Ethiopia, only 7% of children aged 6–23 months met the minimum acceptable diet [12]. This is an alarming gap; still all children have the need and right to sufficient food to support life; there is limited evidence that shows an association between CF indicators with household food security status in Shashemene city, Oromia, Ethiopia. Thus, this study is designed to assess the association between CF practices and household food security status of children aged 6–23 months in the study setting (Figure 1).

### 1.1. Operational Definitions

Timely introduction of complementary feeding: the proportion of children aged 6–23 months who were introduced to solid and semisolid foods at 6 months of age [13, 14].

Minimum dietary diversity (MDD) is the proportion of children of 6–23 months of age who receive foods from 4 or more food groups with the food groups consisting of (I) grains, roots, and tubers; (II) legumes and nuts; (III) dairy products; (IV) flesh foods; (V) eggs; (VI) vitamin A-rich fruits and vegetables; and (vii) other fruits and vegetables during the previous day of study [13, 14].

Minimum meal frequency (MMF) is the proportion of breastfed and nonbreastfed children of 6–23 months of age, who receive solid, semisolid, or soft foods (but also including milk feeds for nonbreastfed children) the minimum number of times or more during the previous day [13, 14].

The minimum is defined as 2 times for breastfed infants of 6–8 months, 3 times for breastfed children of 9–23 months, and 4 times for nonbreastfed children of 6–23 months [13, 14].

A minimum acceptable diet (MAD) is the proportion of children of 6–23 months of age who receive both MMF and MDD during the previous day of study [13, 14].

## 2. Methods and Materials

### 2.1. Study Settings and Period

The research was carried out in Shashemene City from February to March 2020. Shashemene is the most densely populated city in the region, with a diverse ethnic population. It is 250 kilometers from Finfinnee, the capital of Oromia, Ethiopia. Shashemene city is located in the subtropical climatic zone: Shashemene's population is estimated to be 272193 people: in 2019, males accounted for 50.4 percent of the population, while females accounted for 49.6 percent. According to the Shashemene health office report, children aged 6–23 months made up 4.8 percent of the population, or 13065 people.

### 2.2. Study Design and Population

A community-based cross-sectional study was conducted in Shashemene city, Oromia, Ethiopia, from Feb to March in 2020. All mothers who had children aged 6–23 months residing in Shashemene city by 2020 considered as source population whereas mothers of children aged 6–23 months living in selected households during the study period and resided in the study area for more than 6 months were taken as the study population.

### 2.3. Inclusion and Exclusion Criteria

All mother-child pairs aged 6–23 months living in Shashemene city were taken as source population, whereas mother-child pairs aged 6–23 months living in the selected household during the study period as well as resided in the study area for ≥6 months presented during the study period were included as study population while those resided in the study area for <6 months were excluded from the study subjects.

### 2.4. Independent and Dependent Variables

The outcome variable was the CF indicators (timely introduction of CF, MMF, MDD, and MAD). The independent variables were maternal, child, and household characteristics. Briefly, the description of the variables was as follows: sociodemographic characteristics: age, sex, family size, monthly income, partner's education level, and household food security were measured with the Household Hunger Scale (HHS), household wealth index, food insecurity, occupation, residence, knowledge, and attitude of mothers, Obstetric history: pregnancy history, ANC, PNC, delivery mode and place of delivery, birth space, and number of parity) were the independent variables.

As per the WHO definitions already given in the introduction, children receiving the recommended feeding were coded 1 and all other children were coded 0. These are the food groups used in constructing the MDD and MAD, with data on feeding obtained through 24-hour recalls by mothers: grains, roots, and tubers; legumes and nuts; dairy products (milk, yogurt, and cheese); flesh foods (meat, fish, poultry, and liver/organ meats); eggs; vitamin-A-rich fruits and vegetables; and other fruits and vegetables [14]. Household food security was measured with the Household Hunger Scale (HHS) that has 9 items along with 9-frequency (9I 9 F) Household Food Insecurity Access Scale (HFIAS) [15].

### 2.5. Sample Size Determination

The sample size was determined by a single population proportion formula taking the proportion of appropriate complementary feeding practice 11.4% from the previous study [16]. The following assumptions were used: margin of error = 4%, *Zα* = 1.96, and design effect = 2.243; a total of 536 samples mothers were obtained with consideration of 10% contingency to nonresponders.

(1) $$\begin{array}{c} n=\frac{\left(z\alpha /2^{2}\left(1-p\right)\right)}{d^{2}}\gg \frac{{\left(1.96\right)}^{2}*0.114\left(1-0.114\right)}{{\left(0.04\right)}^{2}}=243,243*2+48=536. \end{array}$$

### 2.6. Sampling Procedure

The study subjects were chosen using a two-stage sampling technique. A simple random sampling method was used to select four subcities at random from a total of eight subcities. The total population in the four selected subcities, Buchanan, Arada, Alelu, and Awasho, was 36877, 34529, 31734, and 36370 (139510), with 6696 children aged 6–23 months. The calculated sample (536) was divided equally among the four subcities, resulting in 134 mothers with children aged 6–23 months. To obtain individual sample units or subjects at the household level, all target groups of the subcity were obtained from the health post prior to calculating *K*th and then determining *K*.

**Figure 2:**
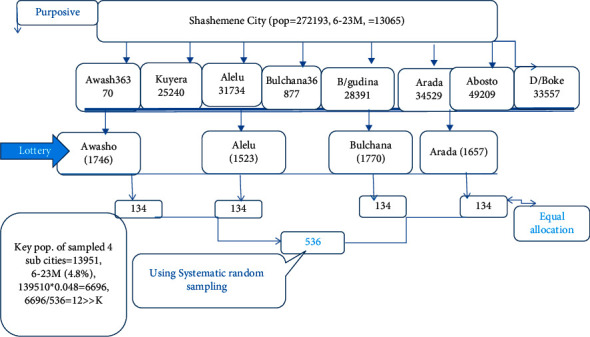
Diagrammatic presentation of the sampling scheme of the study participants among 6–23-month-old children in Shashemene city, Ethiopia, 2020. Pop: population, *K*: the interval.

The random start was determined by lottery, and every *K*th mother with eligible children was chosen from four subcities using systematic random sampling; thus, a child was chosen in each subcity, and his or her mother was interviewed accordingly. One eligible child with a mother at the time of the survey was chosen from each household; if more than two eligible children were found, the younger was chosen, and the process was repeated until the next *K*th in the same direction. If the mother was not present on the day of data collection, she was replaced by the next mother from the same subcity after one visit. It was summarized below (Figure 2).

### 2.7. Data Collection Instruments

Data were collected by using face-to-face interviews during a home-to-home visit from mothers who had children aged 6–23 months using a structured questionnaire. The questionnaire comprised background information on individual characteristics: mothers' age, education, employment status, obstetrics, maternal health practice, children's, age, CF feeding practices, BF, and household characteristics: wealth index, income, and food security/insecurity issues.

### 2.8. Data Collection Methods

Data was collected by using face-to-face interviews during a home-to-home visit from mothers who had children aged 6–23 months using a structured questionnaire. For data collection, first of all, we were collected information about dietary diversity (MDD) meal frequency and MAD of children by using the 24-dietary recall. Socioeconomic and demographic characteristics of children were collected. The four main complementary feeding indicators (timely introduction of CF, MMF, MDD, and MAD), and food security items were included in the questionnaire via 24-hour dietary recalls of food and liquid consumption during the prior day of the survey [13].

Six diploma holder data collectors and two BSc holder supervisors were recruited. For data quality control, the questionnaire was first developed in English and translated to the local language, Afaan Oromo, and then backtranslated to English by two people, who have good command in both languages for consistency. The training was given to data collectors and supervisors for 2 days and the questionnaire was pretested in 5% of mothers, in the study area, which is not included in the actual study to assess the content and approach of the questionnaire and the necessary correction was made. All questionnaires were checked on daily bases for completeness while data was thoroughly checked and cleaned before analysis.

### 2.9. Data Processing and Analysis

Data were coded, entered, and analyzed using SPSS version 25. Descriptive statistics such as frequencies, proportions, means, and standard deviation are used to describe data. Bivariate analysis was made to describe the relation of each independent variable with the dependent variable. Finally, independent variables associated during bivariate analysis with *P*-value ≤0.25 were entered into multivariable logistic regression analysis used to determine the strength of association between independent and dependent variables. OR along with 95%, CI was reported, and the statistical significance was declared at the *p*-value <0.05. Multivariate logistic regression model was used to control confounders.

CF indicators were compared with household food security: Wealth index was computed as a measure of household wealth using principal component analysis (PCA). Fifteen variables related to ownership of selected household assets, size of quantity of durable equipment, materials used for housing construction, and ownership, improved water, and sanitation facilities were considered. Finally, the generated principal component was divided into 5 equal quintiles (lowest, second, middle, fourth, and highest) while household food security was measured with the Household Hunger Scale (HHS) which has 9 items along with 9-frequency (9I 9 F) Household Food Insecurity Access Scale (HFIAS) [15].

The response categories are never (0 times), rarely (1-2 times), or sometimes (3–10 times), and often (more than 10 times). Therefore, the HHS was used in this study to define two groups: households reporting (a) little to no hunger in the past month because of insufficient food or because of lack of resources to get food and thereby classified as food secure households and (b) moderate-to severe hunger in the past month because of insufficient food or because of lack of resources to get food, and thereby classified as food-insecure households.

## 3. Results

### 3.1. Sociodemographic Characteristics of Study Subjects

In this study from 536 sampled mothers, 520 mother-child pairs participated in the study giving the response rate of 97.01%. The mean age of mothers was 26.83 SD (±4.41) years. More than half of the study subjects were between the ages of 25 and 34 years. The most common source of drinking water in study areas is an improved drinking water which piped into the dwelling, a public tap or standpipe 505 (97.1%), the rest 15 (2.9%) from bottled water. Regarding educational status, about 22% of mothers had basic education only whereas 14.8% percent of the households has no work. Of the total study subjects, 481 (92.5%) were married, live at a different place and not married each 14 (2.7%), divorced 10 (1.9%), 1 (.2%) widowed, and 325 (62.5%) Muslim by religion. With regard to ethnic distribution, nearly 2/3, 333 (64%) of the respondents, belong to Oromo ethnic groups. Regarding household food security, out of 520 respondents 407 (78.3%) of children's mothers were under the household who met food security whereas 113 (21.7%) were reported to have had insecure food in the two-week period before the survey (Table 1).

**Figure 3:**
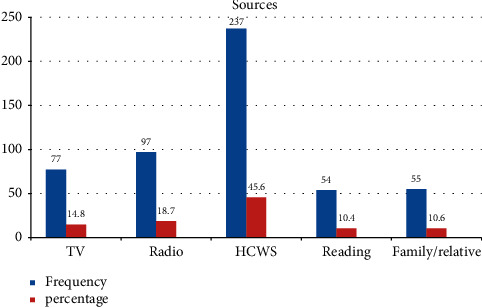
Sources of information about advantages of breast milk feeding in Shashemene city, Oromia, Ethiopia, 2020. TV: television, HCWS: health care workers.

The most common sources of information for the mothers of children aged 6–23 months were health care workers (HCWS) followed by radio (Figure 3). Most study participants 351 (67.5%) were multiparous and of 520 respondents the highest proportion of women 494 (95%) received ANC from a skilled attendant; of these, 41% of mothers have used four or more antenatal care services. Out of 520 respondents, only 288 (55.4%) of them were used postnatal care services. Four hundred fifty (86.5%) of the respondents' birth interval from previous birth were greater than 2 years; with regard to sex, results have shown 262 (50.4%) of the children were male while 258 (49.6%) of them were females.

Concerning delivery attendants, and place of delivery, 426 (81.9%) of mothers were delivered at health facility compared to 94 (18.1%) of home delivery, while 460 (88.5%), 35 (6.7%), and 25 (4.8%) of mothers delivered their children with the assistance of HCWs, TBAs, and others, respectively. Relating to postnatal care, a large proportion of maternal and neonatal deaths occur during the 48 hours after delivery, and these first two days following delivery are critical for monitoring complications. The level of postnatal care coverage is extremely low in this study setting. The great majority of women 231 (44.4%) with a live birth in the preceding survey did not receive a postnatal checkup while among women who received a postnatal checkup, 128 (24.6%) within 0–2 days, 127 (24.4%) within 3–6 days of delivery, and 34 (6.5%) received a postnatal checkup within 7 days and above.

Out of 520 respondents' children aged 6–23 months, 292 (56.2%), 239 (46%), and 329 (63.3) of them experienced symptoms of acute respiratory infection (ARI), diarrhea, and fever, respectively, in the two weeks preceding the survey (Table 2).

**Figure 4:**
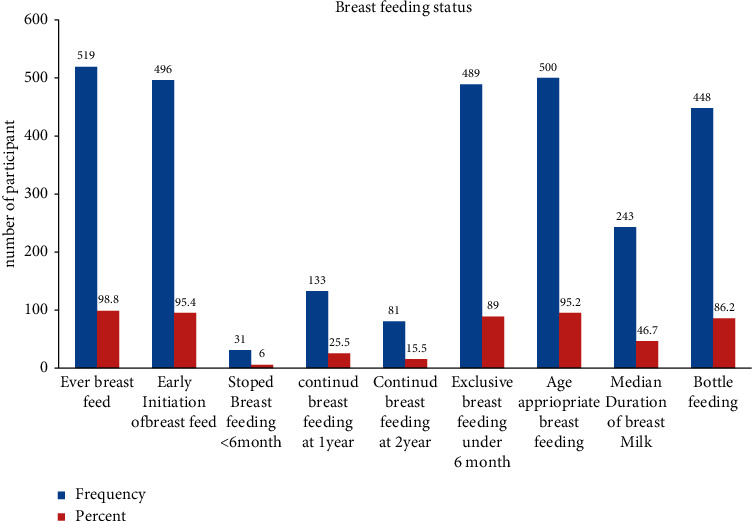
Mothers' breastfeeding status indicators of 6–23-month children in Shashemene Oromia, Ethiopia, 2020. BF: breastfeeding. EBF: exclusive breast feeding.

Almost all mothers 519 (99.8%) BF their children after delivery indicate that very young children are mostly fed breast milk, as recommended by WHO. Most of them 482 (92.7%) feed breast milk based on child demand. The median duration and the mean duration of any BF in Ethiopia are 25 months. In this study, result indicates mothers' BF status; the median duration of BF was 14 months (i.e., 243/520 (46.7%) lower than the EDHS, 2011) (Figure 4).

### 3.2. Food and Fluid Provided for the Children

Milk (417 (80.2 percent) was the most commonly provided food or fluid for children, followed by potatoes (324 (62.3 percent), porridge/gruel 235 (45.2 percent), and bread, vegetables, and fruits 181 (34.8 percent). The most preferred food for the children was homemade, accounting for 398 (76.5 percent), while commercially available food was preferred by the remaining 122 (23.5 percent). In terms of CF practices and frequency during illness, 187 (36%) mothers decreased food quantity and frequency, 147 (28.3%) withheld food quantity and frequency, 123 (23.7%) mothers maintained the same quantity and frequency, and only 63 (12%) mothers increased food quantity and frequency. Due to cultural practices, approximately 124 (23.8%) of mothers did not provide cabbage and meat to their children between the ages of 6 and 23 months. Concerning food preparation, out of 520 mothers, 452 (86.9%) prepare separately for a child while 68 (13.1%) prepared with adult food.

Grains, roots, and tubers were the most commonly taken food items followed by dairy products, among all age categories children in the previous 24 hr preceding the survey. Legumes and nuts, eggs, and vitamin A-rich fruit and vegetables were consumed higher among children aged 18–23 months compared to the other groups in 24 hr preceding survey. Eggs were the least consumed in 6–11-month age group; however, there was less consumption of flesh food across all age categories in contrary to other food groups (Table 3).

### 3.3. Complementary Feeding Status' Indicators

CF was assessed on five hundred twenty mothers-child pairs enrolled in the study. Of these, three hundred fifty-three (67.9%) of the mothers had been introduced for CF at 6 months' age of the children as per suggested while 31 (6%) before 6 months and the rest late after 6 months. Introduction of solid, semisolid, or soft foods by age 6–8 months was 86/93, (92.5%) but vary across age from 112/209 (53.6%) of 12–17 m, −53/70 (75.7%) of 9–11 m, 12–15 m (68.9%), and 18–23 m (68.9%). Overall, 321 (61.7%) mothers fed their children meeting MMF as recommended. Less than half 41.7% of children in all age groups met the criteria for MAD. The lowest proportion who met MAD was (31.2%) in the age group of 6–9 months. However, overall, less than half (42.5%) and 156 (30%) of children aged between 6 and 23 months met the requirements for MDD and ACFP, respectively. Chi-square illustrations of CF indicators and food insecurity are shown in Table 4.

93 (89.2%) mothers fed their child one or two times among breastfed child 6–8 months' age whereas 313 (73.3%) for breastfed child 9–23 months of age fed three or more times.

### 3.4. Bivariate and Multivariable Analyses

Tables 5 and 6 showed bivariate and multivariate associations of household food security with complementary feeding indicators, with Table 5 illustrating factors associated with CF among mothers with children aged 6–23 months. To account for potential confounders, multivariable logistic regression was used. Age, education, occupation, household income, child age, sex, wealth index, food security status, and household monthly food expenditure were the variables.

In this study, there was no association between maternal occupation, maternal education, age 25–29 years, child sex, and CF indicators. Of the total entered variables, the finding revealed socioeconomic status (wealth index, household food security, household income, and child age) are factors statistically significantly associated with the CF indicator, while the rest of the variables were not associated after controlling for potential confounders though associated in bivariate analyses.

Table 5 revealed that there is no association between food security (secure vs. total insecurity) and the introduction of complementary feeding practices at six months (*P*=0.45), but there are associations between food security and MMF (0.002), MDD (*P*=0.03), and MAD (*P*=0.02). There is a relationship between food security and insecurity and their sub groups (food insecure without hunger (*P*=0.23), food insecure with moderate hunger (*P*=0.46), and food insecure with severe hunger (*P*=0.08)) among MMF CF indicators. Aside from that, the table revealed no link between household food insecurity without hunger and MAD (*P*=0.21) or the timely introduction of CF (*P*=0.67).

However, there is an association between household food insecurity without hunger with MDD (*P*=0.006), timely introduction of CF (*p* ≤ 0.001) with moderate hunger, and food insecure with severe hunger (*P*=0.02). However, there was an association between household food security with moderate hunger and MDD (*P*=0.003) and MAD (*P*=0.01) as well as food insecure with severe hunger and MDD (*P*=0.001), MAD (*P*=0.002), and ACFP (*p* ≤ 0.001). Household socioeconomic status (wealth index) was found to be statistically significantly associated with complementary feeding practices. Those mothers who are in highest percentile were about 7, 3.7, and 3.8 times more likely to practice MMF, MDD, and MAD compared to the others counterparts (those who were in lowest percentile through fourth) [(AOR = 6.54, 95% CI: (1.84–20.3)]*∗∗*, [AOR = 3.7, 95% CI: (1.03–13.36)] *∗*, and [AOR = 3.8,95% CI: (1.05–13.73)] *∗*, respectively. After controlling for potential confounders, our results reveal that mothers who belonged to the household whose food security met were 2 times higher to practice MMF compared to the other counterparts [(AOR = 2.02, 95% CI: (1.25–3.24*∗∗*)], *P*=0.04). Others with children in age categories 18–23 months showing a significant association in practicing CF indicators compared with the other groups of children (i.e., MMF [AOR = 5.6, 95% CI: (2.35–13.51) *∗∗∗*, MDD [AOR = 2.31, 95% CI (1.3–4.08), MAD [AOR = 2.18, 95% CI (1.24–3.87), and introduction of CF [AOR = 5.89, 95% CI: (2.49–13.93)]*∗∗∗*) (Tables 5 and 6).

## 4. Discussion

A study was conducted on the association of CF practices and household food security status among mother-child pairs of 6–23 months, which had a response rate of 97.01%. Overall, the result revealed that the magnitude of ACFP was 30%. This is higher than that of the studies done on Damot Sore 11%, Arsi Nagele, 9.5%, and Ghana's 14.3% [16–18], but lower than that of Sri Lankans 68%, Bangladeshi 40%, and Nepali 32% [19, 20]. This is possibly due to differences in the study setting, socioeconomic status, or indicators used to measure appropriate complementary feeding or sociocultural variances among different populations at different times.

In a recent study, the lowest proportion of children who had been fed with ACFP was in the age group of 6–11 months compared with the counter group. This implies the need to give due attention at a younger age. In this study, the proportion of children who had been introduced to solid, semisolid, or soft foods among those aged 6–23 months was 67.9%, while that of 6–8 months was 86/93, or 92.5%.

This is higher than the 74.2% reported by Damot Sore, 72.5% by Arsi Nagele, 72.6% by Ghana, and 70–71.5% reported by Nepali and Bangladeshi studies [16–20]. This figure corresponds to the WHO recommendation that more than 80% of 6–8-month-old children begin complementary feeding at 6 months [13, 14]. Nevertheless, in the current finding, the time of introduction of complementary feeding is better than other similar studies conducted elsewhere [17, 18, 21].

The proportion of children aged 6–23 months who met the MMF criteria was 61.7%. It is comparable to the findings of Arsi Nagele (67.3%) and the Bale Zone Ethiopians (68.4%) [17, 22]. However, it is lower than studies conducted in Sri Lanka (88.3%), Bangladesh (81%), Nepal (82%), coastal South India (77.5%), Derashe, southern Ethiopia (95%), and Amibara district, north east Ethiopia (69.2%) [19, 20, 23–25]. This disparity might be due to the sociocultural, educational, and working conditions of caregivers.

With regard to minimum dietary diversity (MDD), the current study also revealed that the proportion of children who met MDD was 42.5%; this reflects the fact that only these mothers fed their young children with four or more food groups from seven food sets (i.e., grains, roots, and tubers; legumes and nuts; dairy products; flesh foods; vitamin A-rich food; eggs; and other fruits and vegetables) which is almost similar with that of Bangladesh 42%, but higher than the figures stated from studies done in Arsi Nagle 18.8%, Damot sore 16%, India 15%, and Nepal 34%; however, it is lower than that of Sir Lanka 71% [16, 17, 19, 20, 26]. While in Eastern and South Africa it was one in ten infant and young children [27].

The high variation from Damot Sore and Arsi Nagele could be due to the fact that the current study was done in the city where there was better information and maternal health care access, which led to a difference in awareness of the mothers and educational status variations, whereas the previous studies were done in rural areas where mothers were less advantageous compared to their urban counterparts. The low consumption of protein-rich foods can be due to a number of factors, including lack of nutritional awareness and a shortage of access due to economic constraints [28]. The percent study revealed the varieties of foods given to younger children are lower and MDD only tends to increase with growing age (Table 3); that is, the lowest proportion who met MDD was found in the age group of 6–11 months. Similar patterns have also been observed in Ethiopia and other developing countries [17, 29–31].

This might be because mothers may assume that younger infants do not need diversified food or that their guts may not be able to digest animal-source foods. Besides this, the most commonly restricted foods are meat and cabbage, which is why mothers believe that children cannot swallow them. Moreover, flesh food is the least consumed food across all ages, while eggs are the least consumed in the age group 6–11 months. Subsequently, CF might be initiated with monotonous staples. This is in line with a study from northern Ethiopia, which found that flesh foods and eggs were introduced in children's diets in the middle of the second year of age [31]. The study observed that household economic status as measured by wealth index and food security level was a significant predictor of MDD. Obviously, the lower economic status restricts the availability and variety of food in the household.

The minimum acceptable diet incorporates MMF and MDD, which was 41.7%. This is comparable with findings from Bangladesh of 40% and Ghana of 46% where MAD is adopted [18–20]. But this is higher than that of Ethiopia's national level of 7%, and Abiy Adi, north Ethiopia's 11.9%, India's 9%, and Nepal's 32%. But it was lower than the finding from Sri Lanka at 68% [12, 19, 20, 31]. The percent study revealed better conditions in practicing CF indicators than studies done in Damot sore, Arsi Nagele, Abiy Addi ciy, north Ethiopia, and India [16, 17, 31]. Surely, this is the result of health services provided like health education to people in the community. However, 64.5% of infants of 6–8 months of age received the introduction of CF at 6 months of age in 2017, while global rates of MMF, MDD, and MAD were low, at 50.3%, 28.2, and 15.9%, respectively [32].

The lower level of the result could be attributed to socioeconomic, cultural, and policy differences between the study areas as well as time. Thus, the low prevalence of this indicator suggests that the majority of children were either not fed as frequently as the recommended 2–4 times daily or were not offered food from four or more of the recommended food groups in their diet. This may have resulted in inappropriate CF practices which led to malnutrition. High MAD in this study was observed as compared to the national figure of 7% as of EDHS 2016. This might be because EDHs were a nationally representative survey with a wide range of child feeding styles in different parts of the regions of Ethiopia, with a diversified sociocultural context. Besides this, the DHS covers both rural and urban areas, which reduces the figure.

The higher figure observed in our study may be due to the current expansion of HEWs in the study area that focused on ANC, PNC, and child care education, which in turn increases maternal exposure to healthcare workers, thus increasing their practices. Moreover, this is a pocket study that is localized into Ciy where there is better access and availability to information, education, healthcare services, and other social services across time variation. Overall, the proportion of children who met ACFP was 30%. This was higher than that of the studies done on Damot sore, 11.4%, Arsi Nagele, 9.5%, and Abiy Addi Ciy, north Ethiopia, where ACFP was 10.5% [16, 17, 31]. This could be due to differences in the study setting; for example, the previous study was conducted in rural areas of the country where access to maternal health care services and media is limited.

### 4.1. Factors Associated with Complementary Feeding Indicators

The current study revealed that household socioeconomic status (wealth index and food security status, household income) and child age are factors significantly associated with ACFP while the rest of the variables were not associated or lost association after controlling for potential confounders though associated in bivariate analyses. One more important determinant factor associated with CF indicators was household economic status as measured by wealth index and food security level. The household wealth index was found to be statistically significantly associated with CF practices. Those mothers who are in the highest percentile were about 6 times, 3.7 times, and 3.8 times, more likely to practice MMF, MDD, and MAD than those who were in the lowest percentile [(AOR = 6.54, 95% CI: (1.84–23.3)*∗∗*, (AOR = 3.7, 95% CI: (1.03–13.36)*∗∗*, and (AOR = 3.8, 95% CI: (1.05–13.73)]*∗∗*, respectively, but no association with the introduction of CF (0.37, 95% CI: (0.10–1.25), *P*=0.11). So, this illustrates that economically higher people are more likely to practice CF indicators than their counterparts. In fact, a higher household wealth index is positively associated with higher dietary diversity. Families in the high percentile are more likely to be able to afford and offer a variety of foods to their children more frequently. The positive association between a household with the highest wealth percentile and an increased diet diversity has been consistently reported in previous studies done in Sri Lanka, Pakistan, India, Bangladesh, and Nepal [19, 20], which was consistent with our findings.

The 2018 Global Nutrition Report also revealed that the diets of infants and young children are suboptimal everywhere in all wealth groups, from 75.6% in the lowest to 56.7% in the highest quintile [11]. The fact that household wealth is a predictor of MDD underlines the important role of household assets in determining optimal CF practices [19, 20], which is in line with our findings. This study contradicted the findings of Damot Sore and Arsi Nagele, as well as Ethiopia, Nepal, and Sri Lanka, in which maternal education was found to be a predictor of ACFP [16, 17, 19, 20, 31]. The possible explanations can be the difference in societal norms and cultures, with a geographic difference regarding female education.

Child age is also found to be a predictor variable as older children (18–23 months) are about five times more likely to feed CF indicators compared with younger children (6–11 months). Similarly, studies conducted in five Asian countries and Tanzania, Arsi Nagele, and the northern part of Ethiopia reported child age as a predictor variable [17, 19, 20, 27, 32]. This might give an opportunity for the health planners to give due attention to younger children's feeding.

Global nutrition report indicated 74.6% of children 6–23 months of age do not have sufficient diet diversity for a healthy diet worldwide; hence, inappropriate CF after 6 months of age is one major cause of malnutrition; in turn, malnutrition is the leading cause of the global burden of the disease, so attention should be given to young child nutrition education intervention to meet SDG Agenda refers to ending “all forms of malnutrition” [11].

The most important issue regarding feeding during illness was that only 63 (12.3%) of the mothers increased food quantity and frequency. Despite cultural and social food restrictions, commonly on cabbage and meat, which need due attention. Interestingly, there are encouraging practices detected in this study: almost all mothers (519) breastfeed their children after delivery, 496/520 (95.5%) initiate breast feeding earlier, and most of them (482) breast milk based on child demand, whereas 489 (94%) feed EBF versus 58% national Figure of 2016 EDHS. It is recommended that a child continues to breastfeed until the age of two. However, in Ethiopia, the percentage of children who are currently breastfeeding decreases from 91% among children aged 12–17 months to 76% among children aged 18–23 months as of EDHS 2016. That is a bit comparable to this finding.

Homemade food was the most commonly preferred food for the children among 398 (76.5%), while the remaining 122 (23.3%) did not. The most common reasons for early initiation of CF were mothers' negative attitude towards the quantity of breast milk, not staying with the child, and lack of knowledge about breast milk. Nearly 488 (86.2%) mothers used bottle feeding, whereas during illness, only 63 (12%) mothers increased food quantity and frequency, while the rest 88% did not. Despite cultural and social food restrictions on cabbage and meat, which account for 124 (23%), 452 (86.9%) prepared food separately, while 68 (13.1%) were prepared with adult food.

Overall, a low level of ACFP, MDD, and MAD was observed in this study, particularly among children of 11 months of age. Therefore, it needs mitigation to improve CF.

## 5. Limitations

The limitation of our study was it does not demonstrate a cause-and-effect relationship and it may not provide a complete picture of the areas or zones. Hence, our study was conducted among the residents of a single city, which was not representative of the region. Thus, generalizing the result may distort the results to other setting, so caution is needed. Furthermore, the figure could have been overestimated or underestimated due to recall and social desirability biases introduced by the time of initiation, diversity, and frequency of food. The 24-hour dietary diversity recall may only show the most recent feeding and necessitates multiple measurements.

## 6. Conclusion and Recommendation

As a result, the overall CF indicators were low, negatively impacting the health of infants and young children. This demonstrates the significance of taking immediate action to promote CF indicators. Despite the timely introduction of CF, the proportion of mothers whose children met MMF, MDD, and MAD criteria remained a public health challenge in the study area. Child age and household socioeconomic status (food security, wealth index, and income) were among the factors found to be significantly associated with CF indicators.

### 6.1. Recommendation

It was stated in previous studies that two-thirds of child's death was attributed to inappropriate CF practices, in this study CF indicators remaining low. So, to scale up successful interventions to levels that would make an impact, Health Bureau, NGOs, and other development sectors should give special attention to:

- Educational/counseling intervention on nutrition for mothers and/or caregivers is essential for improving infant and young child feeding practices, particularly for mothers with younger children about, time, variety, quantity, and frequency of food
- Promoting socioeconomic status of the community, particularly for poor mothers with households with the lowest wealth index/food insecure via multiple discipline/intersect oral collaboration engagement to improve ACFP
- To come up with the real figure, large-scale longitudinal study will be proposed for the researcher

## Figures and Tables

**Figure 5:**
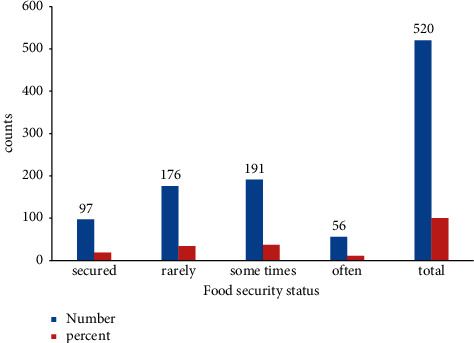
Household food security status among mothers of 6–23 months of age in Shashemene city, Oromia, Ethiopia, 2020. *Note.*Figure 5 indicates household food security status among mothers of 6–23-month children in Shashemene city.

**Table 1:** Sociodemographic characteristic of mothers with children of 6–23 months, in Shashemene city, Oromia, Ethiopia, 2020 (*n* = 520).

Age of mothers/caretakers	Frequency	Percent
≤24 years	151	29.4
25–29 years	231	44.4
30–34 years	100	19.2
35+	38	7.3

Religion of mothers		
Orthodox	95	18.3
Protestant	81	15.5
Catholic	13	2.5
Others	6	1.2

Educational status of mothers		
Basic education	117	22.5
Primary school	236	45.4
Secondary school	113	21.7
Higher education	54	10.4

Educational status of fathers		
Basic education	71	13.7
Primary school	189	36.3
Secondary school	192	36.9
Higher education	68	13.1

Mothers'/caretakers' occupation		
Housewife	244	46.9
Daily labor	150	28.8
Government employee	64	12.3
Students	18	3.5
Merchant	44	8.5

Husband's occupation		
Government employee	149	28.7
Farmer	77	14.8
Merchant	217	41.7
Daily laborer	28	5.4
Others	49	9.4

Family income per month (ETB: Ethiopian birr)		
≤999 ETB	13	2.5
1000–1999 ETB	84	16.2
2000–2999 ETB	129	24.8
3000–3999 ETB	89	17.1
≥4000 ETB	205	39.4

Ethnicity		
Oromo	333	64
Amhara	89	17.2
Wolaita	47	9
Others	51	9.8

Family size		
1–3	101	19.4
4–6	327	62.9
≥7	92	17.7

Who decide on the properties of the household		
Husband	166	31.9
Wife	31	6
Jointly	323	62.1

Sources of information about commercially available CF		
HCWs	95	18.3
Family	163	31.3
Media	215	41.3
Relative	16	6.9
Others	11	2.1

Household wealth index		
Lowest	13	2.5
Second	61	11.7
Middle	203	39
Fourth	151	29

Highest CF: complementary feeding 92; HCWs: Health care workers 17.7.

**Table 2:** Maternal obstetric related characteristics of mothers of children of 6–23 months, Shashemene city, Oromia, Ethiopia, 2020.

Parity	Frequency	Percent
Prim parous (1)	92	17.7
Multiparous (2–4)	351	67.5
Grand multipara (5+)	77	14.8

Mode of delivery		
Normal	468	90
Cesarean section	49	9.4
Others	3	0.6

No antenatal care (ANC) attendance		
No ANC session	26	5
≤3	279	53.7
≥4	215	41.3

Birth spacing		
<2	338	65
2–4	160	30.8
>4	22	4.2

No. of children in the family		
1–3	384	73.8
4–6	120	23.1
>6	16	3.1

Birth order		
1st	101	19.4
2nd	141	27.1
3rd	152	29.2
4th	59	11.3
5th & above	67	12.9

Age of the child		
6–8 months	93	17.9
9–11 months	70	13.5
12–17 months	209	40.2
18–23 months	148	28.5

Mothers' breastfeeding status.

**Table 3:** Types of food groups given during the previous day according to the age of the child among children aged 6–23 months, Shashemene, 2020 (*n* = 520).

Food groups	Age of children in months											
	6–11 months (163)				12–17 months (209)				18–23 months (148)			
	Yes	*N* (%)	No	No (%)	Yes	*N* (%)	No	*N* (%)	Yes	*N* (%)	No	*N* (%)
Grains, roots, and tubers	135	**82.8**	28	17.2	187	**89.5**	22	10.5	131	**88.5**	17	11.5
Legumes and nuts	65	**39.5**	98	60.1	94	**45**	115	55	84	**56.8**	64	43.2
Dairy products	107	**65.6**	56	34.4	139	**66.5**	70	33.5	91	**61.5**	57	38.5
Egg	71	**43.6**	92	56.4	110	**52.6**	99	47.4	90	**60.8**	58	39.2
Flesh foods	20	**12.3**	143	87.7	39	**18.7**	170	81.3	39	**26.4**	109	73.6
Vit. A-rich foods	90	**55.2**	73	44.8	116	**55.6**	93	44.5	108	**73**	40	27
Other fruits and vegetables	77	**47.2**	86	52.8	75	**35.9**	134	64.1	73	**49.3**	75	50.7

Vit. A: Vitamin A.

**Table 4:** Chi-square association of CF indicators with food security status among mothers of 6–23-month children in Shashemene city, Oromia, Ethiopia, 2020 (*N* = 520).

Variables	MMF		MDD		MAD		ACFP		Introduction of CF	
	Met, *N* (%)	Pv	Met, *N* (%)	Pv	Met, *N* (%)	Pv	Met, *N* (%)	Pv	Yes, *N* (%)	Pv
Food secure	71(73.2)	1	47(48.5)	1	47(48.5)	1	40(41.2)	1	66(68)	1
FI without hunger	113(64.2)	0.23	71(40.3)	0.006	69(39.2)	0.21	53(30.1)	0.56	128(72.7)	0.67
FI with moderate hunger	102(53.4)	0.46	69(36.1)	0.003	68(35.6)	≤0.010	35(18.3)	≤0.001	130(68.1)	≤0.001
FI with severe hunger	41(73.2)	0.08	34(60.7)	≤0.001	33(58.9)	0.002	28(50)	≤0.001	29(51.8)	0.02

FI: food insecurity, Pv: *p*-value, CF: complementary feeding, MMF: minimum meal frequency, MDD: minimum dietary diversity, MAD: minimum acceptable diet, ACFP: appropriate complementary feeding, and *N*: count (%).

**Table 5:** Bivariate association of food security status with CF indicators of children aged 6–23 months old in Shashemene city, Oromia, Ethiopia, 2020.

Background characteristics	MMF			MDD			MAD			Introduction of CF		
	COR (95% CI)		Pv	COR (95% CI)		Pv	COR (95% CI)		Pv	COR (95% CI)		Pv
Characteristics of children												
Sex												
M	0.91	0.64–1.30	0.62	0.82	0.57–1.16	0.26	0.87	0.61–1.24	0.44	1.06	0.74–1.54	0.73
F	1			1			1			1		
Child age (in months)												
6–8	1			1			1			1		
9–11	0.93	0.50–1.8	0.93	2.08	1.09–3.96	0.02	1.85	0.97–3.53	0.059	3.94	1.53–10.13	0.004
12–17	0.52	0.31–0.88	0.01	1.31	0.78–2.21	0.31	1.31	0.78–2.21	0.30	10.64	−4.70–24.08	≤0.001
18–23	1.24	0.70–2.18	0.45	2.59	1.50–4.47	0.001	2.45	1.43–4.24	0.001	5.54	2.37–12.90	≤0.001
Characteristics of mothers												
Mothers' age (years)												
≤24	0.80	0.45–1.41	0.45	0.38	0.22–.66	0.001	0.40	0.24–0.70	0.001	0.18	0.07–0.42	≤0.001
25–29	0.78	0.42–1.48	0.43	0.80	0.45–1.42	0.45	0.75	0.42–1.34	0.33	0.71	0.37–1.35	0.30
30–34	0.42	0.27–0.67	≤0.001	0.50	0.33–.77	0.002	0.53	0.34–0.81	0.004	1.92	1.23–2.98	0.004
35+	1			1			1			1		
Mothers' education												
No formal education	1			1			1			1		
Primary school	0.97	0.61–1.54	0.92	1.23	0.78–1.94	0.35	1.21	0.77–1.92	0.39	1.30	0.80–2.10	28
2 yr and above	0.98	0.60–1.61	0.94	1.11	0.68–1.80	0.66	1.15	0.71–1.87	0.56	1.07	0.64–1.80	0.78
Mother's employment												
Homemakers	1			1			1			1		
Unskilled worker	0.67	0.46–0.98	0.04	1.16	0.80–1.68	0.43	1.12	0.76–1.62	0.56	1.57	1.05–2.33	0.02
Skilled workers	0.70	0.39–1.23	0.21	1.51	0.87–2.63	0.14	1.54	0.89–2.68	0.12	1.29	0.71–2.33	0.40
Household characteristics												
Households monthly expenditure on food												
≤3999	1			1			1			1		
≥4000	1.70	1.17–2.47	0.005	2.20	1.54–3.15	≤0.001	2.17	1.52–3.12	≤0.001	0.93	0.64–1.36	72
Households food security status												
Secure	2.07	1.29–3.31	0.002	1.57	1.04–2.40	0.03	1.64	1.08–2.50	0.02	0.84	0.53–1.32	0.45
Insecure	1			1			1			1		
Wealth quintile												
Lowest	1			1			1			1		
Second	1.05	0.32–3.51	0.93	0.49	0.13–1.90	0.30	0.49	0.12–1.90	0.31	0.55	0.17–1.86	0.34
Middle	1.76	0.57–5.41	0.32	1.32	0.39–4.43	0.65	1.29	0.38–4.34	0.68	0.38	0.12–1.19	0.09
Fourth	1.77	0.56–5.52	0.32	2.05	0.61–6.94	0.24	1.89	0.56–6.41	0.30	0.41	0.13–1.29	0.12
Highest	7.09	2.05–24.45	0.002	4.02	1.15–14.07	0.02	4.02	1.15–14.08	0.02	0.30	0.09–.99	0.04

**Table 6:** Multivariate association of food security status with CF indicators of children aged 6–23 months in Shashemene city, Oromia, Ethiopia, 2020.

Background characteristics	MMF			MDD			MAD			Introduction of CF		
	AOR (95% CI)		Pv	AOR (95% CI)		Pv	AOR (95% CI)		Pv	AOR (95% CI)		Pv
Characteristics of children												
Sex												
M	0.91	0.64–1.30	0.61	0.81	0.57–1.16	0.26	0.87	0.61–1.23	0.44	1.05	0.73–1.54	0.78
F	1			1			1					
Child age (months)												
6–8	1			1			1			1		
9–11	3.89	1.46–10.37	0.007	2.21	1.12–4.37	0.02	1.94	0.98–3.85	0.05	4.11	1.57–10.73	0.004
12–17	11.34	4.87–26.39	≤0.001	1.43	0.82–2.48	0.19	1.42	0.83–2.46	0.20	11.18	4.88–25.61	≤0.001
18–23	5.64	2.35–13.51	≤0.001	2.31	1.3–4.08	004	2.18	1.24–3.87	0.007	5.89	2.49–13.93	≤0.001
Characteristics of mothers												
Age (yrs)												
≤24	0.60	0.29–1.43	0.28	0.91	0.43–1.91	0.81	0.86	0.41–1.81	0.69	5.63	1.89–16.76	0.002
25–29	0.68	0.28–1.32	0.21	0.92	0.45–1.87	0.82	0.90	0.44–1.84	0.78	4.45	1.56–12.68	0.005
30–34	1.07	0.39–2.14	0.84	1.52	0.70–3.29	0.28	1.47	0.68–3.18	0.32	2.46	0.80–7.53	0.11
35+	1			1			1			1		
Mothers' education												
No formal education	1			1			1			1		
Primary school	1.02	0.62–1.66	0.93	1.17	0.73–1.8	0.49	1.05	0.63–1.77	0.82	1.3	0.80–2.21	0.72
2 yr and above	0.91	0.53–1.54	0.72	0.92	0.55–1.53	0.73	1.24	0.80–1.91	0.32	1.0	0.62–1.88	0.78
Mother's employment												
Homemakers	1			1			1			1		
Unskilled worker	1.65	0.91–3.01	0.09	0.84	0.47–1.50	0.57	0.82	0.46–1.48	0.52	0.88	0.47–1.66	0.70
Skilled workers	1.23	0.67–2.4	0.49	1.04	0.57–1.87	0.21	0.96	0.53–1.72	0.89	1.13	0.60–2.13	0.69
Household characteristics												
Household's monthly expenditure on food												
≤3999	1			1			1			1		
≥4000	1.72	1.16–2.53	0.006	2.29	1.58–3.33	≤0.001	2.25	1.55–3.25	≤0.001	0.91	0.61–1.37	0.67
Households food security status												
Secure	2.02	1.25–3.24	0.004	1.55	1.02–2.36	0.04	1.62	1.06–2.47	0.02	0.87	0.55–1.39	0.58
Insecure	1			1			1			1		
Wealth quintile												
Lowest	1			1			1			1		
Second	1.00	0.29–3.42	0.99	0.45	0.12–1.79	0.26	0.46	0.12–1.82	0.27	0.64	0.18–2.21	0.48
Middle	1.68	0.53–5.31	0.37	1.23	0.36–4.20	0.74	1.22	0.35–4.18	0.75	0.44	0.14–1.39	0.16
Fourth	1.71	0.53–5.48	0.36	1.89	0.55–6.56	0.31	1.78	0.51–6.16	0.36	0.46	0.14–1.48	0.19
Highest	6.54	1.84–23.3	0.04	3.70	1.03–13.36	0.46	3.80	1.05–13.73	0.04	0.37	0.10–1.25	0.11

## Data Availability

Data used to support the study are available from the corresponding author upon request.

## Abbreviations

ACFP: Appropriate complementary feeding practice
ANC: Antenatal care
AOR: Adjusted odds ratio
C/S: Caesarian section
CF: Complementary feeding
CI: Confidence interval
COR: Crude odds ratio
CSA: Central statistical agencies
EDHS: Ethiopian Demographic and Health Survey
EBF: Exclusive breast feeding
BF: Breast feeding
FI: Food insecurity
HCWs: Health care workers
IYCF: Infant and young child feeding
MAD: Minimum acceptable diet
MDD: Minimum dietary diversity
MMF: Minimum meal frequency
OR: Odds ratio
NGOs: Nongovernmental organizations
PPS: Probability proportionate to size
PNC: Postnatal care
SDGs: Sustainable development goals
SPSS: Statistical Package for the Social Sciences software
TBAs: Traditional birth attendants
UNICEF: United Nation International Children's Emergency Funds
WHO: World Health Organization.
